# Influence of dietary energy level and corn particle size on performance, nutrient digestibility, and gut development in broilers fed pelleted diets

**DOI:** 10.1016/j.psj.2026.107447

**Published:** 2026-07-16

**Authors:** Mikael Sihite, Mohammad R. Abdollahi, Nicola M. Schreurs, Birger Svihus, Timothy J. Wester

**Affiliations:** a School of Agriculture and Environment, Massey University, Palmerston North, 4442, New Zealand; b Department of Animal Science, Tidar University, Magelang, 56116, Indonesia; c Department of Animal and Aquacultural Sciences, Norwegian University of Life Sciences, Ås, 1433, Norway

**Keywords:** Energy level, Low AMEn, High AMEn, Particle size

## Abstract

This study evaluated the effects of dietary energy levels and corn particle sizes on the growth performance and nutrient digestibility of broiler chickens. The energy used in this experiment is based on nitrogen-corrected apparent metabolizable energy (AMEn): low AMEn (2,850 kcal/kg) and high AMEn (3,000 kcal/kg) were paired with three corn particle sizes: 2.5, 5.0, and 8.0 mm. Birds fed low-energy diets had significantly greater feed intake and weight gain (*P* < 0.01). Coarse particles had the lowest FCR when diets contained high AMEn (*P* < 0.05), while the lowest FCR in the low AMEn group was produced by medium particles (*P* < 0.05). The CAID of DM, GE, and P were increased by feeding fine particles in the low AMEn group (*P* < 0.05). Greater CAID of starch and N was found in birds fed low-energy diets (*P* < 0.05). The absolute and relative weight of breast meat was greater in the low AMEn group (*P* < 0.05). Energy levels or particle sizes did not significantly affect the weights and lengths of the small intestine compartments (*P* > 0.05). A heavier gizzard was found in birds fed coarse particles (*P* < 0.05), as well as the lowest gizzard pH (*P* < 0.05). Treatments did not affect the villi length of the jejunum and ileum, and crypt depth of the jejunum (*P* > 0.05), but affected the ileum crypt depth (*P* < 0.05). Also, no effect on jejunum and ileum epithelial thickness (*P* > 0.05). The findings indicate that the benefits of coarse particles on gizzard weight and function are evident, although enhancements in the AIDE and CAID of DM, GE, and P occurred only when coarse particles were combined with high-energy diets. The performance of birds was most notable on a low-energy diet, likely due to increased feed intake.

## Introduction

To enhance performance and improve efficiency in poultry production, modification of grain as the primary energy source has been extensively studied. Decreasing energy levels result in reduced body weight gain (Leeson et al., 1996; Massuquetto et al., 2020). Increasing dietary energy levels results in excessive fat deposition, particularly in the abdominal fat pad, reducing feed efficiency and carcass yield (Mabray and Waldroup, 1981; Deaton et al., 1983; Mendes et al., 2004) and has been related to birds’ health issues, such as sudden death syndrome (Mollison et al., 1984). Continuous genetic progress in broiler strains has led to changes in the nutrient balance of broilers. This idea was reviewed by Aftab (2019) who proposed modern broilers could tolerate lower energy, as Chang et al. (2015) reported that feeding a lower energy level (92.5% of the recommended level) is not detrimental to body weight. Classen (2017) stated that dietary energy is one of the factors that affect birds’ performance, including the diet's structure and quality, which further affects feed intake.

The particle size of the feed also influences the efficiency of nutrient utilization in birds. Increasing the particle size of grains in pelleted diets has been the subject of several reports, revealing potential benefits for the poultry industry (Lentle et al., 2006; Amerah et al., 2007b; Naderinejad et al., 2016; Svihus et al., 2024a). Coarser particle sizes, achieved through larger grinding screen sizes, not only reduce energy consumption during feed processing but also improve gizzard function (Svihus, 2011; Abdollahi et al., 2019). The impact of coarser particles on gut development and health has also been reviewed by Zaefarian et al. (2016), who highlighted that improved digestion results from enhanced gut function, as shown by increased villus height and a reduction in pathogenic bacteria due to the lower pH in the gizzard. The gizzard, which is essential for mechanical digestion in birds, benefits from larger particle sizes, as it leads to increased gizzard weight due to increased muscle capacity, enhanced gizzard function, and increased nutrient digestibility and absorption (Svihus et al., 2004; Svihus, 2011; Amerah et al., 2008; Pacheco et al., 2013; Kheravii et al., 2017; Marx et al., 2021; Rueda et al., 2024). However, some reports are conflicting, showing no improvement in digestibility, even though gizzard weight increased (Jacobs et al., 2010; Tejeda and Kim, 2021).

Energy levels and particle size influence the quality of the diet and further influence nutrient utilization. Greater energy diets have been reported to have a lower pellet quality (Reece et al., 1986; Pope et al., 2020), and the detrimental effects of coarser particles on pellet quality have also been reported (Zang et al., 2009; Córdova-Noboa et al., 2021; Svihus et al., 2024a). However, da Silva et al. (2018) and Naderinejad et al. (2016) reported a relatively high pellet quality in diets made of coarse particles (applying up to 6.5 and 8 mm screen, respectively). This inconsistency highlights the need to investigate the interaction between energy levels and particle size on the quality of both diets and the performance of birds. Diets with fine particle sizes may result in less efficient digestion due to reduced gizzard function, as Naderinejad et al. (2016) reported a lower AME in birds fed finer particles associated with decreased gizzard function in pellet feeding. In contrast, coarser particle sizes may enhance gizzard function, facilitating a more effective mechanical breakdown of the feed, resulting in improved energy digestion and absorption.

As Chang et al. (2015) reported that feeding energy lower than recommended (−200 kcal) was not detrimental to broilers' performance, the interaction between energy levels and particle size has not been evaluated. Earlier studies by Reece et al. (1985) and Lott et al., 1992 used different mills to alter particle size and energy levels over 3,000 kcal but did not report effects on nutrient utilization, diet quality, or gut development. The present experiment aimed to determine broilers’ performance, nutrient digestibility, breast meat weight, and gut development when fed different particle sizes (fine, medium, and coarse from 2.5-, 5.0-, and 8.0 mm screens, respectively) with low- and high-energy AMEn-based diets.

## Materials and methods

The experiment reported in this study was conducted in accordance with the Massey University Animal Ethics Committee guidelines (MUAEC 24/44).

### Experimental diets

This experiment used a 2 × 3 factorial arrangement of treatments to evaluate two energy levels and three corn particle sizes. The standard energy level of 3,000 kcal is considered a high-energy diet, and the 2,850 kcal is considered a low-energy diet. The energy levels are formulated based on the apparent metabolizable energy corrected for nitrogen (AMEn). Three particle sizes of corn for fine, medium, and coarse were obtained by grinding corn with 2.5, 5.0, and 8.0 mm screens in a hammer mill. Whole corn grains were obtained from a local commercial supplier.

Diets were formulated to be equal to the nutritional requirements of the Ross 308 strain (Aviagen, 2022). The diets were formulated to meet the required nutrient specifications using Allix3 feed formulation software (version 25.06; A-Systems, Versailles, France). The calculated composition of the diets is presented in Table 1. The high AMEn treatment was obtained by the inclusion of a greater quantity of soybean oil, and the low-energy (−150 kcal/kg) diet was obtained by reducing oil utilization as an expensive energy source ingredient in broilers’ diets. In the diets, 5.0 g/kg of titanium dioxide (TiO₂, Merck KGaA, Darmstadt, Germany) was used as an indigestible marker to determine ileal nutrient digestibility. Diets for the experiment were pelleted using a pellet mill (Model Orbit 15; Richard Sizer Ltd., Kingston-upon-Hull, UK), with a 3-mm die ring featuring 3-mm holes and a 35-mm thickness.

**Table 1:** Calculated composition and analysis of the experimental diets (g/kg as fed).

Item	Energy level	
	Low AMEn	High AMEn
Corn	570.9	536.1
Soybean meal	373.9	381.2
Soybean oil	12.6	40.6
DL-Methionine 99.0%	3.2	3.0
L-Lysine HCl 79.8%	2.0	1.9
L-Threonine 98.5%	1.3	1.2
L-Isoleucine 98.0%	0.7	0.6
L-Valine 96.5%	0.5	0.5
Dicalcium phosphate	11.7	11.7
Limestone	9.8	9.8
Sodium bicarbonate	3.9	3.8
Vitamin and Mineral premix¹	2.0	2.0
Sodium chloride	1.7	1.8
Choline chloride 60.0%	0.7	0.7
Titanium dioxide	5.0	5.0
Phytase	0.1	0.1
Price USD/T	$860.3	$909.4
Calculated Analysis		
AMEn, kcal/kg	2,850	3,000
Crude Protein	205	205
Digestible methionine	6.67	6.70
Digestible methionine + cysteine	9.62	9.60
Digestible lysine	12.70	12.70
Digestible threonine	8.45	8.47
Digestible arginine	13.51	13.62
Digestible valine	9.52	9.52
Digestible histidine	4.94	4.94
Digestible tryptophane	2.25	2.27
Digestible isoleucine	8.41	8.41
Digestible leucine	16.92	16.80
Digestible tyrosine	7.37	7.41
Digestible phenylalanine	9.70	9.72
Crude fat	36.85	63.41
Crude fiber	11.30	10.61
Total calcium	9.50	9.50
Analyzable Calcium	8.00	8.00
Non-phytate phosphorus	5.00	5.00
Sodium	2.00	2.00
Chloride	2.00	2.00
Potassium	10.82	10.89

¹Vitamin and trace mineral premix supplied the following per kilogram of diet: antioxidant, 100 mg; biotin, 0.2 mg; calcium pantothenate, 12.8 mg; vitamin D3 (cholecalciferol), 2400 IU; cyanocobalamin, 0.017 mg; folic acid, 5.2 mg; menadione, 4 mg; niacin, 35 mg; pyridoxine, 10 mg; vitamin A (trans-retinol), 11,100 IU; riboflavin, 12 mg; thiamine, 3.0 mg; vitamin E (dl-α-tocopherol acetate), 60 IU; choline chloride, 638 mg; Co, 0.3 mg; Cu, 3.0 mg; Fe, 25 mg; I, 1 mg; Mn, 125 mg; Mo, 0.5 mg; Se, 200 µg; Zn, 60 mg.

### Birds and housing

A total of 288, one-day-old male broiler chicks (Ross 308), obtained from a commercial hatchery, were weighed and allocated to 36 floor pens (1.6 × 0.8 m) in a room heated by electric heaters. Each of the dietary treatments was then randomly assigned to six of the pens, with eight birds in each pen.

The birds were transferred to wired cages (0.6 × 0.6 m) at the end of the second week for excreta collection on days 18 to 21. The floor pens and wired cages were housed in an environmentally controlled room, which provided 20 hours of fluorescent illumination per day. The temperature was maintained at 31°C on day one and was gradually reduced to 22°C by 21 days of age. The experimental diets were offered *ad libitum* from the 1st to the 21st day, and water was always available. Body weight (BW) to determine weight gain (WG), feed intake (FI), and feed conversion ratio (FCR) were recorded, and the calculation of the FCR was conducted with a correction for mortality during the trial.

### Particle size distribution and pellet quality

The dry sieving method was employed to determine the particle size distribution of the ground corn and mash diet (before pelleting) using the procedure of Baker and Herrman (2002). Particle size distribution analysis of the diets was conducted using wet sieving according to the method described by Lentle et al. (2006). Briefly, a 100 g portion was dried at 80°C in a forced draught oven for three days to ascertain the dry matter (DM) content, while another 100 g was soaked in 400 mL of water and left for two hours before sieving. The samples were then washed, using 4 L of water over five minutes, through a set of six steel sieves with apertures of: 2,000, 1,000, 500, 250, 125, and 63 μm (Endecotts Ltd., London, UK). After sieving, the contents of each sieve were dried on pre-weighed filter papers in a fan-forced oven at 80°C for 24 hours and re-weighed. The total DM recovered was calculated as the total dry weight of particles retained on each sieve. The geometric mean diameter (GMD) and geometric standard deviation (GSD) of the ground corn and diets were subsequently determined based on the equations below, assuming that the weight distribution is logarithmically normal according to the method of Baker and Herrman (2002).

$$d_{i}={\left(d_{u}\times d_{o}\right)}^{0.5}$$

$$\text{GMD}=\log^{-1}\left[\frac{\sum \;\left(W_{i}\log d_{i}\right)}{\sum W_{i}}\right]$$

$$\text{GSD}=\log^{-1}{\left[\frac{\sum \;W_{i}\;{\left(\log d_{i}-\log\text{GMD}\right)}^{2}}{\sum \;W_{i}}\right]}^{0.5}$$

Where,

$d_{i}$: diameteroftheithsieveonthestack.
$d_{u}$: diameteroftheithsieveonthestack.
$d_{o}$: diameteroftheithsieveonthestack.

Intact pellet testing used 100 g of feed sample and was conducted using the Endecotts sieve shaker (Endecotts Ltd., London, UK), with two screens of 2,000 and 1,000 μm. The weight of the intact pellets retained on the 2,000 μm sieve, the fines retained on the 1,000 μm sieve, and the fines that passed through the 1,000 μm sieve were recorded after a two-minute test cycle.

The pellet durability index (PDI) was determined by the percentage of the retained pellet in the test chamber after a 30-second test cycle in an air stream at 70mBar using a Holmen Pellet Tester (New Holmen NHP100 Portable Pellet Durability Tester, TekPro Ltd., Willow Park, North Walsham, Norfolk, UK), following the method by Abdollahi et al. (2010). Pellet hardness was evaluated using a Kahl pellet hardness tester.

### Excreta preparation for apparent metabolizable energy (AME) and apparent ileal digestible energy (AIDE) analysis

The total collection of excreta to determine the apparent metabolizable energy was conducted on days 18 to 21. The feed intake and total excreta output for each replicate were recorded over the final four days. Daily excreta collections were pooled within replicates. Excreta obtained over four days were homogenized using a blender to prepare the subsample and then lyophilized for each replicate. Diets and dried excreta were ground to pass through a 0.5 mm sieve and stored in airtight containers at 4°C prior to analysis. Both ground excreta and diets were analyzed for dry matter (DM), nitrogen (N), and gross energy (GE).

### Coefficient of apparent ileal digestibility determination

The collection of digesta from the ileum was performed on day 21. A total of six birds from each replicate were euthanized using sodium pentobarbital injection (0.5 ml/kg BW) in the lower wing veins. The small intestine was exposed by making an incision above the cloaca. Ileal digesta was collected by flushing the distal ileum with distilled water into plastic containers. The ileum was identified as the lower part of a bird’s small intestine, extending from Meckel’s diverticulum to 40–50 mm from the ileocecal junction. Digesta samples were lyophilized and ground to pass through a 0.5 mm sieve, then stored at 4°C prior to analysis of DM, N, fat, starch, titanium (Ti), P, and GE.

### Breast meat and fat pad weight

Breast meat and fat pad weight were measured from two additional birds (apart from birds that were euthanized for ileal collection). The two birds were euthanized by sodium pentobarbital injections in the lower wing veins. Skinless breast meat (pectoralis major and minor) was removed from the sternum, weighed, and calculated as g/kg BW. The fat pad was accessed by an incision to the abdominal cavity, removed, and weighed relative to live body weight (g/kg BW).

### Digestive tract, gizzard pH, jejunum, and ileum morphology measurement

Two digestive tracts were taken from the birds used for breast meat and fat pad measurement. The body weight was recorded. The gut was freed from the mesentery and sectioned at the junction of the proventriculus with the gizzard, at the junction of the gizzard with the duodenum, at Meckel’s diverticulum, and 1 cm above the ileocecal junction (the junction of the small intestine with the colon immediately proximally to the caeca) to obtain the proventriculus, gizzard, duodenum, jejunum and ileum, and caeca respectively. The length of each segment was measured using a flexible measuring tape before the contents were removed. After the contents were removed, flushed with water, dried with tissue paper, and weighed. The gut compartments were determined as the relative length (cm/kg BW) and weight (g/kg BW).

Gizzard pH was measured using two gizzards from two birds euthanized for ileal collection using the method described by Singh et al. (2014). The measurement was conducted in three different regions (proximal, middle, and distal) by inserting a calibrated pH meter (Model IQ120, ISFET pH Meter, Shindengen, Japan). Gizzard pH was the average of the three readings (proximal, middle, and distal) from each gizzard.

Jejunum and ileum sections were taken for intestinal morphological examination. Each section was approximately 5 cm long, flushed with cold saline, and immediately placed in a container containing 70% formalin. After 72 hours, the samples were transferred to 70% ethanol. They were drained, cut to approximately 8 mm, placed in the cassette, and then processed in a tissue processor (ThermoFisher Excelsior ES, Waltham, State). The samples were dehydrated through graded alcohols (70%, 95%, and absolute alcohol) at ambient temperature, cleared in isopropyl alcohol, and impregnated with Histosec pastilles using pressure at 60°C. The samples were embedded in wax and cut using a rotary microtome (MicroTec, Walldorf, Germany) with Feather S35 disposable blades at a thickness of 5 µm. They were then stained with alcian blue and hematoxylin-eosin before being examined by light microscopy. Two segments were fixed on each slide, and the slides were observed using an Olympus microscope (BX51TF, Olympus, Tokyo, Japan). The variables measured for the jejunum and ileum included villus height (the distance from the apex of the villus to the junction of the villus and crypt), crypt depth (the distance from the junction to the basement membrane of the epithelial cell at the bottom of the crypt), and epithelial thickness (the distance from the epithelial surface to the basement membrane of the epithelial cell). Visual measurements of villus height were conducted on 10 villi at 4 × magnification, 10 × for crypt depth, and 40 × for epithelial thickness. The measurement process was conducted using computer software (cellSens Dimension, Olympus, Tokyo, Japan).

### Chemical analysis

Dry matter was determined using the oven-drying method (Methods 930.15; AOAC, 2016). Nitrogen was analyzed through combustion using a carbon nanosphere-200 auto analyzer (rapid MAX N exceed, Elementar, Donaustraze, Hanau, Germany) (Method 968.06; AOAC, 2016). The content of crude protein (CP) was calculated as N × 6.25. Gross energy was measured using an adiabatic bomb calorimeter (Gallenkamp Autobomb, London, UK) standardized with benzoic acid. Crude fat was determined by the Soxtec extraction procedure (Method 2003.06; AOAC, 2016) employing the Soxtec System HT 1043 Extraction Unit, Sweden. Total starch content was quantified using a Megazyme kit (Method 996.11; AOAC, 2016) based on thermostable α-amylase and amyloglucosidase. Ti in the diets and digesta were analyzed on a UV spectrophotometer (Short et al., 1996).

### Calculations

All data were expressed on a DM basis. The AME was determined by the following formula:

$$\text{AME}\;\text{Diet}\;\left(\text{MJ}/\text{kg}\right)=\frac{\left[\left(\text{FI}\;\times \;\text{GE}\;\text{Diet}\right)-\;\left(\text{Excreta}\;\text{output}\;\times \;\text{GE}\;\text{Excreta}\right)\right]}{\text{FI}}$$

The calculation of nutrients’ coefficient of apparent ileal digestibility (CAID) was from the dietary ratio of nutrients to titanium dioxide (Ti) relative to the corresponding ratio in the ileal digesta.

$$\text{CAID}\;\text{of}\;\text{diet}\;\text{nutrient}=\;\frac{\left[{\left(\text{Nutrient}\;/\;\text{Ti}\right)}_{\text{diet}}-\;{\left(\text{Nutrient}\;/\;\text{Ti}\right)}_{\text{ileal}}\right]}{{\left(\text{Nutrient}\;/\;\text{Ti}\right)}_{\text{diet}}}$$

Where,

${\left(\text{Nutrient}\;/\;\text{Ti}\right)}_{\text{diet}}$: ratioofnutrienttoTiinthediet,and
${\left(\text{Nutrient}\;/\;\text{Ti}\right)}_{\text{ileal}}$: ratioofnutrienttoTiintheilealdigesta.

### Data analysis

Cage served as the experimental unit for all data, excluding the digestive tract measurement. Individual birds were considered the experimental units for measurements of the digestive tract. Diet prices from local commercial suppliers were converted to USD, and FCR data were used to calculate the feed cost per kg WG for 21 days of the experiment. The experiment was in a 2 × 3 factorial arrangement, and the data were analyzed using two-way ANOVA to determine the main effects (energy levels and particle size) and their interaction, employing the SAS General Linear Models procedure. Differences were deemed significant at *P* < 0.05, and *P* < 0.1 was discussed as a trend. The Least Significant Difference test was employed to identify significant differences between means, with *P* < 0.05 considered significant.

## Results

### Particle size distribution and pellet quality

The pelleting process reduced the proportion of coarse particles in the diets (Table 2). Among all treatments, the greatest PDI was achieved with the low-energy diet and coarse grinding (80%), while the lowest was noted in the combination of high energy and coarse grinding (51%) (Table 3). In low-energy diets, coarse particles did not negatively affect PDI. The detrimental effect of coarse particles on PDI occurred when the diet contained high energy. On average, the PDI of diets formulated to be high-energy was 59% regardless of grinding, whereas for the low-energy diet it was 79%. The greatest pellet hardness was made of medium grinding and low energy (1.07 N), while the lowest pellet hardness was found in a diet made of fine particles combined with high energy (0.95 N) (Table 3).

**Table 2:** Determined particle size distribution (percentage of retained particles on sieves), and geometric mean diameter ± geometric standard deviation (GMD ± GSD, μm) of the ground corn, mash diets, and pellet diets.

Particle size	Opening (μm)							GMD ± GSD
	2,000	1,000	500	250	125	63	<63	
Ground corn¹ (Dry sieving)								
Fine	0.00	21.13	32.44	36.37	6.74	3.32	0.00	540.90 ± 2.00
Medium	12.36	33.28	20.90	17.46	10.80	5.08	0.12	700.36 ± 2.49
Coarse	30.07	26.18	17.31	10.95	10.69	4.60	0.20	859.64 ± 2.64
Mash diets¹ (Dry sieving)								
Low AMEn								
Fine	0.00	24.09	40.71	25.07	10.13	0.00	0.00	610.35 ± 1.90
Medium	8.76	29.03	30.15	22.81	9.25	0.00	0.00	716.53 ± 2.08
Coarse	18.27	27.02	27.04	18.27	9.28	0.12	0.00	809.62 ± 2.19
High AMEn								
Fine	0.00	25.12	50.43	23.42	1.03	0.00	0.00	705.32 ± 1.65
Medium	7.71	28.89	40.06	21.12	2.22	0.00	0.00	789.32 ± 1.84
Coarse	17.70	27.28	30.95	21.79	2.28	0.00	0.00	868.75 ± 1.97
Mash diets² (Wet sieving)								
Low AMEn								
Fine	1.92	27.04	21.01	10.65	6.87	5.41	27.10	317.82 ± 3.94
Medium	14.71	24.63	17.47	8.41	5.52	4.17	25.09	404.65 ± 4.30
Coarse	22.52	23.03	15.78	7.60	4.67	3.15	23.25	476.97 ± 4.38
High AMEn								
Fine	1.52	26.97	22.58	10.51	6.40	5.17	26.85	322.31 ± 3.91
Medium	13.42	26.11	17.80	9.04	6.29	4.60	22.74	418.88 ± 4.15
Coarse	23.01	23.00	15.88	7.97	5.33	3.82	20.99	496.81 ± 4.25
Pellet diets² (Wet sieving)								
Low AMEn								
Fine	0.68	18.29	21.46	11.10	6.91	3.06	38.50	227.22 ± 3.97
Medium	2.76	18.12	18.51	11.99	7.03	3.94	37.65	231.59 ± 4.04
Coarse	4.69	21.25	19.56	10.63	6.84	3.50	33.53	275.99 ± 4.15
High AMEn								
Fine	0.34	5.94	26.79	8.19	6.33	2.75	49.66	159.42 ± 3.64
Medium	2.57	17.50	19.16	11.17	6.84	3.08	39.68	223.66 ± 4.07
Coarse	3.44	16.31	19.41	11.34	7.85	3.50	38.15	227.93 ± 4.03

AMEn is N-corrected Apparent Metabolizable Energy (Low AMEn is 2,850 kcal and High AMEn is 3,000 kcal).

Fine, medium, and coarse grades were achieved using screen sizes of 2.5, 5.0, and 8.0 mm, respectively.

¹Each value represents the mean of five replications.

²Each value represents the mean of two replications.

**Table 3:** Intact pellet, pellet durability index (PDI)%, and pellet hardness (N) of the diets.

Energy levels	Particle size	Percentage of intact pellets and fines¹			Pellet quality	
		Pellets retained (2,000 μm sieve)	Fines retained (1,000 μm sieve)	Fines (<1,000 μm)	PDI²	Pellet hardness³
Low AMEn	Fine	96.1	1.9	2.0	79	1.05
	Medium	97.1	1.2	1.7	77	1.07
	Coarse	96.4	1.7	1.9	80	1.02
High AMEn	Fine	93.8	3.3	2.9	67	0.95
	Medium	94.8	2.9	2.3	59	0.97
	Coarse	94.2	2.1	3.7	51	1.01

AMEn is N-corrected Apparent Metabolizable Energy (Low AMEn is 2,850 kcal and High AMEn is 3,000 kcal).

Fine, medium, and coarse grades were achieved using screen sizes of 2.5, 5.0, and 8.0 mm, respectively.

¹Each value represents the mean of five replicates.

²Each value represents the mean of eight tests.

³Each value represents the mean of 15 tests.

### Performance

Feed intake was affected by energy level (*P* < 0.05). Birds fed a low-energy diet (1,501 g) had a greater intake than those fed a high-energy diet (1,438 g). Birds fed high-energy diets consumed 4.2% less feed (63 g/bird) than those fed low-energy diets. There was no interaction effect of energy levels and particle size on FI (*P* > 0.05). The bird WG was greater (*P* < 0.05) when fed diets with low energy levels (1,265 g), while the WG of birds fed diets with high energy levels was 1,228 g. Neither particle size nor its interaction with energy levels affected WG (*P* > 0.05). There was an interaction of energy levels and particle size on the FCR (*P* < 0.01). In the low-energy diet group, medium particles exhibited a lower FCR (1.179), whereas FCR was similar for fine and coarse particles (1.191 and 1.192; fine to coarse, respectively). In high-energy diets, the lowest FCR was found in birds fed coarse particles (1.160), followed by fine (1.178) and medium particles (1.192). Feed cost was dependent on the energy levels, as there was an interaction of energy levels and particle size (*P* < 0.05). In high-energy diets, the cheapest diet was the one made of coarse particles, and diet prices were similar regardless of particle size in low-energy diets (Table 4)).

**Table 4:** Influence of dietary energy level and particle size on FI (g), WG (g), FCR, and feed cost (USD/kg WG) of 21-day-old broilers¹.

Energy level	Particle size	FI	WG	FCR	Feed cost (USD/kg WG)
Low AMEn	Fine	1,492	1,254	1.191ᵃ	1.028ᶜ
	Medium	1,504	1,278	1.179ᵃᵇ	1.018ᶜ
	Coarse	1,505	1,263	1.192ᵃ	1.029ᶜ
					
High AMEn	Fine	1,432	1,224	1.178ᵃᵇ	1.076ᵃᵇ
	Medium	1,446	1,213	1.192ᵃ	1.088ᵃ
	Coarse	1,435	1,246	1.160ᵇ	1.058ᵇ
SEM^2^		15.8	16.2	0.0068	0.0060
					
*Main effects*					
Energy level					
Low AMEn		1,501ᵃ	1,265ᵃ	1.187	1.025
High AMEn		1,438ᵇ	1,228ᵇ	1.177	1.074
					
Particle Size					
	Fine	1,462	1,239	1.185	1.052
	Medium	1,475	1,246	1.185	1.053
	Coarse	1,470	1,255	1.176	1.044
					
*Probabilities, P≤*					
Energy level		0.001	0.009	0.081	0.001
Particle size		0.714	0.632	0.325	0.275
Energy level × Particle size		0.926	0.333	0.009	0.007

Fine, medium, and coarse grades were achieved using screen sizes of 2.5, 5.0, and 8.0 mm, respectively.

AMEn is N-corrected Apparent Metabolizable Energy (Low AMEn is 2,850 kcal and High AMEn is 3,000 kcal).

Means in a column not sharing common letters (a, b, and c) are different (*P* < 0.05).

¹Each value represents the mean of six replicates (eight birds per replicate).

²Pooled standard error of the mean.

### Coefficient of apparent ileal digestibility and energy digestibility

The CAID of DM, GE, starch, N, fat, phosphorus, AIDE, and the AMEn of birds fed different energy levels and particle sizes are presented in Table 5. There was a significant interaction of energy levels × particle size on the CAID of DM, GE, P, and AIDE (*P* < 0.05). For the high-energy diets, the lowest CAID of DM, GE, P, and AIDE was observed in birds fed fine particles. While in low-energy diets, the greatest CAID of DM, GE, and P was observed in diets with a fine particle size (*P* < 0.05). The CAID of starch and N was affected by energy levels (*P* < 0.05), and greater CAID of starch and N was found in birds fed low-energy diets. The CAID of fat was similar among treatments (*P* > 0.05). For the low-energy diets, the AIDE was greater in birds fed fine particles (*P* < 0.05), whereas the greatest AIDE in high-energy diets was found in those fed coarse particles. The greatest AMEn was found in those birds fed high-energy diets (*P* < 0.05).

**Table 5:** Influence of dietary energy level and particle size on coefficient of apparent ileal digestibility (CAID)¹ of dry matter (DM) (%), starch (%), N (%), fat (%), P (%), and AIDE (MJ/kg DM) and N-corrected apparent metabolizable energy (AMEn; MJ/kg DM)² of 21-day-old broilers.

Energy level	Particle size	CAID						AIDE	AMEn
		DM	GE	Starch	N	Fat	P		
Low AMEn	Fine	0.764ᵃ	0.800ᵃ	0.984	0.851	0.942	0.659ᵇ	14.64ᵃ	12.57
	Medium	0.708ᵇ	0.754ᵇ	0.984	0.834	0.949	0.577ᵃᵇ	13.78ᶜ	12.48
	Coarse	0.712ᵇ	0.760ᵃᵇ	0.985	0.849	0.944	0.595ᵃᵇ	13.89ᵇᶜ	12.53
									
High AMEn	Fine	0.691ᵇ	0.759ᵇ	0.981	0.789	0.932	0.493ᵃ	14.13ᵃᵇᶜ	13.92
	Medium	0.722ᵃᵇ	0.765ᵃᵇ	0.974	0.815	0.927	0.623ᵃᵇ	14.43ᵃᵇ	13.98
	Coarse	0.723ᵃᵇ	0.770ᵃᵇ	0.977	0.833	0.941	0.657ᵇ	14.52ᵃ	14.05
SEM³		0.012	0.010	0.003	0.014	0.007	0.028	0.193	0.286
									
*Main effects*									
Energy level									
Low AMEn		0.728	0.771	0.984ᵃ	0.845^a^	0.945	0.610	14.10	12.53ᵇ
High AMEn		0.712	0.761	0.978ᵇ	0.815^b^	0.933	0.591	14.36	13.98ᵃ
									
Particle Size									
	Fine	0.727	0.775	0.983	0.824	0.937	0.576	14.38	13.25
	Medium	0.715	0.759	0.979	0.825	0.938	0.600	14.10	13.24
	Coarse	0.718	0.765	0.980	0.841	0.943	0.626	14.21	13.28
									
*Probabilities, P≤*									
Energy level		0.115	0.254	0.026	0.015	0.076	0.422	0.110	0.001
Particle size		0.556	0.342	0.565	0.413	0.716	0.228	0.367	0.977
Energy level × Particle size		0.001	0.007	0.634	0.345	0.473	0.001	0.007	0.952

Fine, medium, and coarse grades were achieved using screen sizes of 2.5, 5.0, and 8.0 mm, respectively.

AMEn is N-corrected Apparent Metabolizable Energy (Low AMEn is 2,850 kcal and High AMEn is 3,000 kcal).

Means in a column not sharing common letters (a and b) are different (*P* < 0.05).

¹Each value represents the mean of six replicates (six birds per replicate).

²Each value represents the mean of six replicates (eight birds per replicate).

³Pooled standard error of the mean.

### Breast meat and fat pad

Both the absolute and relative weights of breast meat from broilers on the low-energy diets were heavier than those fed high-energy diets (*P* < 0.05; Table 6). The relative weight of the fat pad was affected by dietary energy levels (*P* < 0.05; Table 6).

**Table 6:** Influence of dietary energy level and particle size on breast meat weight (g) and relative weight to body weight (g/kg BW) of 21-day-old broilers¹.

Energy level	Particle size	Absolute weight		Relative to BW	
		Breast meat	Fat pad	Breast meat	Fat pad
Low AMEn	Fine	276	11.3	190	0.78
	Medium	272	11.3	186	0.78
	Coarse	270	11.0	196	0.79
					
High AMEn	Fine	254	11.8	180	0.84
	Medium	236	12.5	175	0.93
	Coarse	262	12.4	185	0.89
SEM²		9.24	0.74	4.49	0.049
					
*Main effects*					
Energy level					
Low AMEn		273ᵃ	11.2	191ᵃ	0.78ᵇ
High AMEn		250ᵇ	12.2	180ᵇ	0.88ᵃ
					
Particle Size					
	Fine	265	11.6	185	0.81
	Medium	254	11.9	181	0.85
	Coarse	266	11.7	190	0.84
					
*Probabilities, P≤*					
Energy level		0.006	0.099	0.005	0.018
Particle size		0.373	0.907	0.110	0.678
Energy level × Particle size		0.322	0.786	0.973	0.634

Fine, medium, and coarse grades were achieved using screen sizes of 2.5, 5.0, and 8.0 mm, respectively.

AMEn is N-corrected Apparent Metabolizable Energy (Low AMEn is 2,850 kcal and High AMEn is 3,000 kcal).

Means in a column not sharing common letters (a and b) are different (*P* < 0.05).

¹Each value represents the mean of six replicates (two birds from each replicate).

²Pooled standard error of the mean.

### Digestive tract measurement

The proventriculus weight was not affected by energy levels or particle size (*P* > 0.05; Table 7). Gizzards were heavier in birds fed on bigger particle sizes (*P* < 0.05; Table 7). The gizzard's pH was significantly lower in birds fed coarse particle size (*P* < 0.05; Table 7). The relative length of the duodenum, jejunum, ileum, and caeca was similar among treatments (*P* > 0.05; Table 7). There was no effect of energy levels or particle size on the duodenum, jejunum, ileum, and caeca length relative to body weight (*P* > 0.05; Table 8).

**Table 7:** Influence of energy level and particle size on gut compartments’ (g/kg BW)¹ and caeca (g/kg BW)², gizzard weight (g), and pH³ of 21-day-old broilers.

Energy level	Particle size	Proventriculus	Gizzard		Small intestine			Caeca
			Weight	pH	Duodenum	Jejunum	Ileum	
Low AMEn	Fine	3.57	10.5	3.32	4.23	6.60	5.81	2.53
	Medium	3.59	11.3	3.04	4.10	6.86	6.02	2.74
	Coarse	3.54	12.5	2.97	4.26	7.49	6.05	2.73
								
High AMEn	Fine	3.76	10.4	3.46	4.37	7.41	6.41	2.54
	Medium	3.93	11.2	3.20	4.32	7.19	6.13	2.69
	Coarse	3.52	11.3	2.93	4.10	7.97	6.03	2.54
SEM⁴		0.139	0.454	0.131	0.265	0.275	0.283	0.14
								
*Main effects*								
Energy level								
Low AMEn		3.57	11.4	3.11	4.20	6.99	5.96	2.67
High AMEn		3.74	10.9	3.20	4.26	7.19	6.19	2.59
								
Particle Size								
	Fine	3.67	10.4ᵇ	3.39ᵃ	4.30	7.00	6.11	2.54
	Medium	3.76	11.3ᵃᵇ	3.12ᵇ	4.21	7.03	6.07	2.72
	Coarse	3.53	11.9ᵃ	2.95ᵇ	4.18	7.23	6.04	2.64
								
*Probabilities, P≤*								
Energy level		0.155	0.210	0.420	0.772	0.364	0.330	0.498
Particle size		0.277	0.011	0.010	0.886	0.662	0.972	0.448
Energy level × Particle size		0.447	0.450	0.701	0.745	0.065	0.526	0.783

Fine, medium, and coarse grades were achieved using screen sizes of 2.5, 5.0, and 8.0 mm, respectively.

AMEn is N-corrected Apparent Metabolizable Energy (Low AMEn is 2,850 kcal and High AMEn is 3,000 kcal).

Means in a column not sharing common letters (a and b) are different (*P* < 0.05).

¹Each value represents the mean of six replicates (two birds from each replicate).

²Each value represents the mean of six replicates (two birds from each replicate; the weight value from each bird is the average of the left and right caecum weights).

³Each value represents the mean of six replicates (two birds from each replicate; each gizzard was assessed three times: proximal, middle, and distal).

⁴Pooled standard error of the mean.

**Table 8:** Influence of energy level and particle size on small intestine compartments (cm/kg BW)¹ and caeca relative length (cm/kg BW)² of 21-day-old broilers.

Energy level	Particle size	Small intestine			Caeca
		Duodenum	Jejunum	Ileum	
Low AMEn	Fine	22.1	53.3	59.9	12.5
	Medium	22.4	52.9	58.6	12.3
	Coarse	22.4	54.5	59.1	12.6
					
High AMEn	Fine	22.3	54.8	59.1	12.8
	Medium	21.8	53.9	63.9	13.7
	Coarse	21.1	51.1	57.0	11.3
SEM³		0.80	1.76	2.18	0.55
					
*Main effects*					
Energy level					
Low AMEn		22.3	53.6	59.2	12.5
High AMEn		21.7	53.3	60.0	12.6
					
Particle Size					
	Fine	22.2	54.1	59.5	12.6
	Medium	22.1	53.4	61.2	13.0
	Coarse	21.8	52.8	58.1	12.0
					
*Probabilities, P≤*					
Energy level		0.414	0.822	0.660	0.816
Particle size		0.876	0.766	0.357	0.192
Energy level × Particle size		0.660	0.320	0.202	0.070

Fine, medium, and coarse grades were achieved using screen sizes of 2.5, 5.0, and 8.0 mm, respectively.

AMEn is N-corrected Apparent Metabolizable Energy (Low AMEn is 2,850 kcal and High AMEn is 3,000 kcal).

¹Each value represents the mean of six replicates (two birds from each replicate).

²Each value represents the mean of six replicates (two birds from each replicate; the length value from each bird is the average of left and right caecum lengths).

³Pooled standard error of the mean.

### Jejunum and ileum morphology

There was no effect of energy level of the diet and particle size on the jejunum villi length and crypt depth (*P* > 0.05). The villi length of the ileum was not affected by the energy levels and particle size (*P* > 0.05; Table 9). However, a thicker crypt depth of the ileum was found in birds fed diets with a fine particle size (*P* < 0.05). There was no effect of energy levels or particle size on epithelial thickness in both the jejunum and the ileum (*P* > 0.05; Table 9).

**Table 9:** Influence of dietary energy level and particle size on the villi length, crypt depth, and epithelial thickness of jejunum and ileum (µm) of 21-day-old broilers.

Energy level	Particle size	Jejunum			Ileum		
		Villi length¹	Crypt depth²	Epithelial thickness	Villi length¹	Crypt depth²	Epithelial thickness
Low AMEn	Fine	1169	179	48.1	714	141	40.5
	Medium	1115	168	46.2	687	125	40.7
	Coarse	1252	161	47.0	735	121	41.0
High AMEn	Fine	1251	158	46.7	715	131	41.0
	Medium	1281	167	44.3	696	129	41.7
	Coarse	1168	159	46.7	689	120	42.8
SEM³		61.55	9.32	2.27	28.40	6.01	1.19
*Main effects*							
Energy level							
Low AMEn		1179	169	47.1	712	129	40.7
High AMEn		1233	161	45.9	700	127	41.9
Particle Size							
	Fine	1210	169	47.4	714	136ᵃ	40.7
	Medium	1198	167	45.2	691	127ᵃᵇ	41.2
	Coarse	1210	160	46.8	712	121ᵇ	41.9
*Probabilities, P≤*							
Energy level		0.286	0.306	0.520	0.617	0.665	0.206
Particle size		0.975	0.596	0.604	0.675	0.049	0.578
Energy level × Particle size		0.137	0.501	0.940	0.591	0.528	0.822

Fine, medium, and coarse grades were achieved using screen sizes of 2.5, 5.0, and 8.0 mm, respectively.

AMEn is N-corrected Apparent Metabolizable Energy (Low AMEn is 2,850 kcal and High AMEn is 3,000 kcal).

Means in a column not sharing common letters (a and b) are different (*P* < 0.05).

¹Each value represents the mean of observable villi length in stains (two stains per slide).

²Each value represents the mean of the measured crypt depth below the observable villi length.

³Pooled standard error of the mean.

## Discussion

The use of coarse grinding in poultry processing has the potential to reduce energy consumption. It was reported that there is a 35% reduction in energy consumption when using a 7.9-mm screen compared to a 4.76-mm screen in hammermill grinding (Reece et al., 1986). However, there are detrimental effects of coarse particles on pellet durability (Zang et al., 2009; Chewning et al., 2012; Mohammadi Ghasem Abadi et al., 2019; Córdova-Noboa et al., 2021; Svihus et al., 2024a). This current study confirms a decrease in pellet durability with increased coarseness of corn particles. However, this finding is only observed in high-energy diets. Pelleted diets comprising high-energy formulations have lower PDI as reported by Briggs et al. (1999), who found a 60% reduction in PDI (82.0 vs. 49.2%) in diets with greater energy from oil, with the oil content being increased from 3.9% to 7.9%. A similar finding by Pope et al. (2020) reported a 50.47% reduction in PDI when diets included 10 compared to 50 g/kg of oil.

In the current study, pellet durability index (PDI) was similar across diets with different particle sizes that were formulated to be lower energy (less oil content). The most durable pellets were for diets composed of coarse particles (80%). Other studies that utilized different particle sizes also reported similar PDI (Amerah et al., 2007a; Pope et al., 2022). A report by Naderinejad et al. (2016) employed a similar screen grinding method to the present study and reported PDI values of 74.6, 78.8, and 79.9% for 2.0-, 5.0-, and 8.0-mm screens, respectively, from fine to coarse. Therefore, both particle size and energy level affected PDI, but the impact of particle size was dependent on the energy level of the diet. Improved PDI and pellet hardness have been associated with fine particles, as the surface binding area increases (Angulo et al., 1996; Muramatsu et al., 2015). However, this report indicates that the benefits of fine particles on PDI and pellet hardness were diminished if the diets contained high energy. Pellets with high PDI are favored in the feed industry, as birds prefer intact pellets (Nir et al., 1994; Abdollahi et al., 2013; Svihus et al., 2024b). Therefore, this study suggests that if PDI and pellet hardness are considered essential in diets, attention to diet energy levels in formulation is necessary.

The notion that birds can adjust their feed intake based on the energy content in their diets was evident in the current study. Birds fed a low-energy diet had a greater feed intake than those fed a high-energy diet (1,501 vs. 1,438 g). Weight gain was also greater for those birds fed low-energy diets, likely stimulated by the increased FI. A greater feed intake for a dietary energy of 2,800 kcal compared to 3,200 kcal was also observed by Abdollahi et al. (2018). Additionally, Zhang et al. (2024) reported a greater daily FI in birds offered 2,860 kcal compared to those with 2,930 and 3,000 kcal. Similar findings have been found in several studies (Brickett et al., 2007; Wang et al., 2014; Yu et al., 2024). It is noticeable that birds compensate for low-energy diets by increasing their feed intake to meet their nutrient requirements, which indicates the importance of energy to support all physiological functions in poultry, including maintenance, growth, and muscle deposition. It is worth considering that the increased FI in birds fed low-energy diets may also be partially attributed to the improved PDI and pellet hardness. In this experiment, the average PDI of diets with low energy and high energy was 79% and 59%, respectively.

The benefits of coarse particles on birds' FCR have been reported (Amerah et al., 2008; Mohammadi Ghasem Abadi et al., 2019). This study noted the positive impact of coarse particle size on FCR only when birds were offered high-energy diets. In the high-energy diets group, the lowest FCR was observed in birds fed a coarse particle size (1.160), followed by fine (1.178) and medium (1.192). In contrast to those fed low-energy diets, the lowest FCR was found in those fed medium particle sizes (1.179). FCR was observed to improve in those fed high-energy diets, likely because energy requirements for both maintenance and production were met. For those fed a low-energy diet, the benefits of coarse particles were diminished, probably due to birds' need to obtain more energy to meet their energy requirements, not to mention the greater energy needed in the gizzard to grind the coarse particles.

There was a numerical improvement in CAID of DM and GE from fine to medium particles when birds fed high-energy diets. When birds were offered low-energy diets, the CAID of DM and GE was most notable in fine particles. Again, the CAID improvement in those fed high-energy diets is hypothesized because of improved gizzard function, as it improves the digestibility of nutrients (Svihus, 2011). In contrast to the low-energy diet group, the improvement in digestibility was presumably attributed to enhanced digestion and prolonged enzymatic contact resulting from increased FI. Greater FI has been reported to increase nutrient load in the gut and slow the passage rate, hence improving enzymatic digestion (Massuquetto et al., 2020). The improvement in CAID of starch and N was also attributed to the slower passage rate, as they were only significantly improved in those fed low-energy diets. The CAID of fat was found to be the same among treatments, but the tendency of increased CAID of fat (*P* = 0.076) was also found in birds fed low- energy diets. The AIDE also showed the pattern of improved digestibility as mentioned above, while AMEn was significantly greater in birds fed high-energy diets. This has been anticipated, as greater AMEn has been reported in birds fed high-energy diets in some reports (Abdollahi et al., 2018; Khoddami et al., 2018; Hamungalu et al., 2020).

In the present report, the influence of particle size on the CAID of P was dependent on the energy levels. The CAID of P was improved in the coarse particles when birds were offered high-energy diets. While in low-energy diets, CAID of P was most pronounced in fine particles. The prominent effect of the coarse particle in this study is in contrast to Mtei et al. (2019) and Naderinejad et al. (2016), who found similar CAID of P, regardless of particle size in corn-based diets. Mineral digestibility in birds fed coarse particles is hypothesized due to the longer transit time of digesta in the gut (Amerah et al., 2007a). The improvement in P digestibility in birds fed coarse particles has also been reported in some studies using corn-based diets. Kasim and Edwards (2000) reported an improved coefficient of retention of P from 44.4% (484 μm) to 50.6% (894 μm) in broilers, while total P retention improvement from 48.0% (606 μm) to 54.6% (1,094 μm) was reported by Charbeneau and Roberson (2004) in 15-day turkey poults.

Particle size did not affect breast meat weight and its relative weight. Interestingly, breast meat relative to BW was greater in birds fed low-energy diets (190.4 vs. 179.95 g/kg). Reports on the influence of varying energy levels on broiler breast meat have primarily been conducted in older broilers and are mostly limited to those over 35 days, rather than at 21 days of age in the current study. Zhang et al. (2025) investigated a range of energy levels (2,709, 2,976, and 3,225 kcal) in interaction with protein and reported a greater relative weight of breast meat in 62-day-old broilers on low-energy diets. In addition, Toghyani et al. (2025) reduced the energy in diets by 50, 100, and 150 kcal from the recommended energy in 42-day broilers and found a greater breast meat-to-BW ratio in the lower energy diets, specifically 237 vs. 246 g/kg BW for those with the recommended ME and those reduced by 150 kcal, respectively. The increase in breast meat in birds fed low-energy diets has been reported to be an indirect effect of increased FI, as birds obtain more protein to gain muscle (Toghyani et al., 2025). In this current report, FI in birds fed low-energy diets was greater than that in birds fed high-energy diets (1,438 vs. 1,501 g). In addition, the greater oil inclusion in the high-energy diets may have influenced breast muscle accretion through alterations in nutrient partitioning, as dietary lipid level has been reported to modify protein utilization and carcass protein deposition in broilers (Liu et al., 2017). While more protein was obtained with greater intake in low-energy diets, low intake in high-energy diets did not provide more protein; hence, the fat pad was significantly greater in high-energy feeding. As greater fat pad weight has been reported in birds given high-energy diets (Zhai et al., 2014; Zhang et al., 2025), the fat pad deposition is highly influenced by dietary energy levels.

The length and weight of the small intestine were not affected by particle size, as reported in other studies (Péron et al., 2005; Parsons et al., 2006; Amerah et al., 2007b; Fronte et al., 2013; Naderinejad et al., 2016; Mohammadi Ghasem Abadi et al., 2019; Pourazadi et al., 2020) or energy levels (Ivanovich et al., 2017). Dietary energy levels did not affect gizzard weight and pH. However, coarse particles significantly increased gizzard weight, consistent with findings of other studies (Amerah et al., 2008; Fronte et al., 2013; Naderinejad et al., 2016; Mtei et al., 2019; Tejeda and Kim, 2021; Pope et al., 2022). The pelleting process has been known to reduce particle size (Naderinejad et al., 2016), but the current report highlighted that using a larger screen still benefited gizzard function by the coarseness of the particles, as coarser grinding yields more coarser particles even after the pelleting process (Table 2). The gizzard pH was also found to be lower in birds fed a coarse particle size due to the prolonged digestion of coarse particles, resulting in more gastric juice release to aid digestion (Svihus, 2011). It has been reported that the low gizzard pH is associated with gut health (Engberg et al., 2002, 2004; Svihus, 2011). In the current report, the ileum crypt depth was thinner in those fed coarse particles, indicating a healthier small intestine. Crypts are responsible for regenerating new cells for the epithelial cells of the villi (Pashaei Jalal et al., 2024), either as a response to the adverse effect of the presence of pathogenic bacteria (Zaefarian et al., 2016) or damaging substances from the diet. Greater cell turnover from the crypt leads to heavier function, resulting in thicker crypts, and it is hypothesized to occur in the current report. Considering a relatively short passage rate of the feed in a bird's digestive system, it is also hypothesized that epithelial cell replacement occurred more frequently in the distal small intestine, as there was no difference in crypt depth in the jejunum among treatments in this experiment.

This finding shows that both reducing energy levels and coarse grinding can be implemented in the modern broiler industry. From a nutrient utilization perspective, decreasing energy in the diet leads to increased feed intake, hence improved nutrient digestibility and nutrient intake and further improves performance. Improved gizzard function in relation to improved energy digestibility is also presented in this current report in diets made of coarse grinding. On the feed cost side, feeding broilers with energy levels lower than the recommended (2,850 kcal/kg; AMEn-based) reduces feed cost by 4.9 cents per kg of WG. In high-energy diets, the feed cost for diets formulated with coarse particles was also the lowest (3 cents lower) per kg WG than that of diets formulated with medium grinding. Also, coarse grinding is likely to reduce the energy cost of diet preparation during the grinding process (cost saving not determined in this study).

## Disclosures

The authors declare that they have no known competing financial interests or personal relationships that could have appeared to influence the work reported in this paper.
